# Therapeutic effect of acupuncture combined montelukast sodium on cough variant asthma in children: A protocol for systematic review and meta-analysis

**DOI:** 10.1097/MD.0000000000028048

**Published:** 2021-12-23

**Authors:** Xiuling Zhou, Ye Zhang, Le Liu, Xiaochun Feng, Hongshi Zhang

**Affiliations:** a School of Nursing, Changchun University of Traditional Chinese Medicine, Changchun, China; b College of Acupuncture and Tuina, Changchun University of Traditional Chinese Medicine, Changchun, China; c School of Traditional Chinese Medicine, Changchun University of Chinese Medicine, Changchun, China; d Jilin Provincial Hospital of Traditional Chinese Medicine, Jilin, China.

**Keywords:** acupuncture combined, cough variant asthma in children, montelukast sodium

## Abstract

**Background:**

Cough variant asthma in children is a special type of asthma. Although there are many effective cases of combined acupuncture and western medicine in the clinical treatment of this kind of children, there is no standardized acupuncture combined with western medicine to evaluate the curative effect. Therefore, combined with existing reports, a systematic review and meta-analysis of acupuncture combined with montelukast sodium in the treatment of cough variant asthma in children were carried out to obtain conclusive results.

**Methods:**

The following electronic databases will be searched: PubMed, the Cochrane Library, Embase, Web of Science, Medline, CNKI, Chinese Biomedical Literature Database, VIP, and Wan Fang databases. We will consider articles published between database initiation and October 2021. We will use Review Manager 5.4, provided by the Cochrane Collaborative Network for statistical analysis. Clinical randomized controlled trials related to acupuncture combined with montelukast sodium on cough variant asthma in children were included in this study. Language is limited to both Chinese and English. Research selection, data extraction, and research quality assessments were independently completed by two researchers. We then assessed the quality and risk of the included studies and observed the outcome measures.

**Results:**

This study provides a high-quality synthesis to assess the effectiveness and safety of acupuncture combined with montelukast sodium on cough variant asthma in children.

**Conclusion:**

This systematic review will provide evidence to determine whether acupuncture combined with montelukast sodium is an effective and safe intervention for patients with cough variant asthma in children.

**INPLASY Registration number:**

INPLASY2021110006.

## 1 Introduction

Cough variant asthma in children (CVA) is a special type of asthma. Cough is the only or main clinical manifestation.^[1]^ There are no obvious symptoms or signs such as wheezing or shortness of breath, but there is airway hyperresponsiveness. It mostly occurs in preschool and school-age children. According to research, in recent years, the incidence of cough variant asthma in children has shown an upward trend year by year, and it has become a hot research project of pediatric respiratory diseases. As the disease is chronic, persistent, stubborn and refractory, and recurring, it seriously affects the physical and mental health of the children.^[2]^ Although there are many effective cases of combined acupuncture and western medicine in the clinical treatment of this kind of children, there is no standardized acupuncture combined with western medicine to evaluate the curative effect.^[3]^ Therefore, combined with existing reports, a systematic review and meta-analysis of acupuncture combined with montelukast sodium in the treatment of cough variant asthma in children were carried out to obtain conclusive results.

## 2 Methods

### 2.1 Protocol and registration

The agreement has been registered on the Open Science Framework (INPLASY) platform (https://inplasy.com/inplasy-2021–11–0006/), registration number:INPLASY2021110006. This program is the preferred reporting project based on the Guidelines for the Systematic Review and Meta-Analysis Program (PRISMA-P),^[4]^ and the final report will be in line with PRISMA's recommendations on the extended statement of the systematic review report included in the meta-analysis of medical interventions.^[5]^

### 2.2 Eligibility criteria

#### 2.2.1 Type of studies

Any randomized controlled trials (RCTs) exploring the effectiveness and safety of AM for the treatment of patients with CVA will be included. We will not consider other studies, such as non-clinical trials, non-controlled trials, and non-RCTs.

#### 2.2.2 Type of participants

Studies on adult patients, 18 years old or below, who were diagnosed as CVA will be included in this study. No limitations of location, educational background, and sex will be imposed.

#### 2.2.3 Type of interventions

Any forms of AM therapy used to treat patients with CVA will be included in the experimental group. Any other treatments, but not AM, used to manage participants with CVA will be entered in the control group.

#### 2.2.4 Type of outcomes

Comparing the clinical efficacy of the two groups, the criteria for judging: the symptom disappeared after 1 week of treatment and no recurrence within 3 months is markedly effective; after the treatment, the cough symptoms of the child are alleviated, but occasionally it is effective as treatment; Later, the symptoms of the child did not disappear or even worsened to be invalid. The lung function indexes of the 2 groups before and after treatment were compared, including the forced end expiratory volume in the first second (FEV_1_) and the forced vital capacity.^[6]^ The improvement of clinical symptoms in the two groups was compared, including the duration of asthma, wheezing, and time to disappear cough. The inflammatory factor indexes before and after treatment were compared between the two groups, including C-reactive protein and procalcitonin.^[7]^ Observe the occurrence of adverse reactions such as dizziness, headache, abdominal pain, nausea, and vomiting in the 2 groups.

### 2.3 Search strategy and analysis

#### 2.3.1 Electronic searches

The selection of the time for inclusion of the literature will be selected from the establishment of each database to October 30, 2021, by searching PubMed, Embase, Cochrane Library, Chinese Biomedical Literature Database (CBM), Chinese National Knowledge Infrastructure (CNKI), Chinese Scientific Journal Database (VIP), PubMed, and other 7 databases. Keywords include “acupuncture combined montelukast sodium,” “Cough variant asthma,” and so on. Specific search terms in Table 1.

**Table 1:** Search strategy for the PubMed database.

No.	Search terms
#1	Cough variant asthma(all field)
#2	Variant asthma(all field)
#3	Pediatric Asthma(all field)
#4	Pediatric variant asthma(all field)
#5	cough variant asthma(all field)
#6	#1OR#2–5
#7	acupuncture(all field)
#8	acupuncture therapy(all field)
#9	scalp acupuncture(all field)
#10	fire needling(allfield)
#11	Intradermal needling(all field)
#12	ear acupuncture(all field)
#13	acupoint(all field)
#14	auricuar acupuncture(all field)
#15	electroacupuncture(all field)
#16	catgut embedding(all field)
#17	#7 OR #8-16
#18	Montelukast Sodium(all field)
#19	Singulair(all field)
#20	Montelukast(all field)
#21	Montelukast Sodium Tablets(all field)
#22	#18 OR #19-21
#23	randomized controlled trial(all field)
#24	randomly(all field)
#25	controlled clinical trial(all field)
#26	randomized(all field)
#27	random allocation(all field)
#28	single-blindmethod(all field)
#29	double-blindmethod(all field)
#30	trials(all field)
#31	Comparators
#32	allocation
#33	#23 OR #24-32
#34	#6 And #17 And #22 And #33

#### 2.3.2 Data extraction and quality assessment

**Figure 1:**
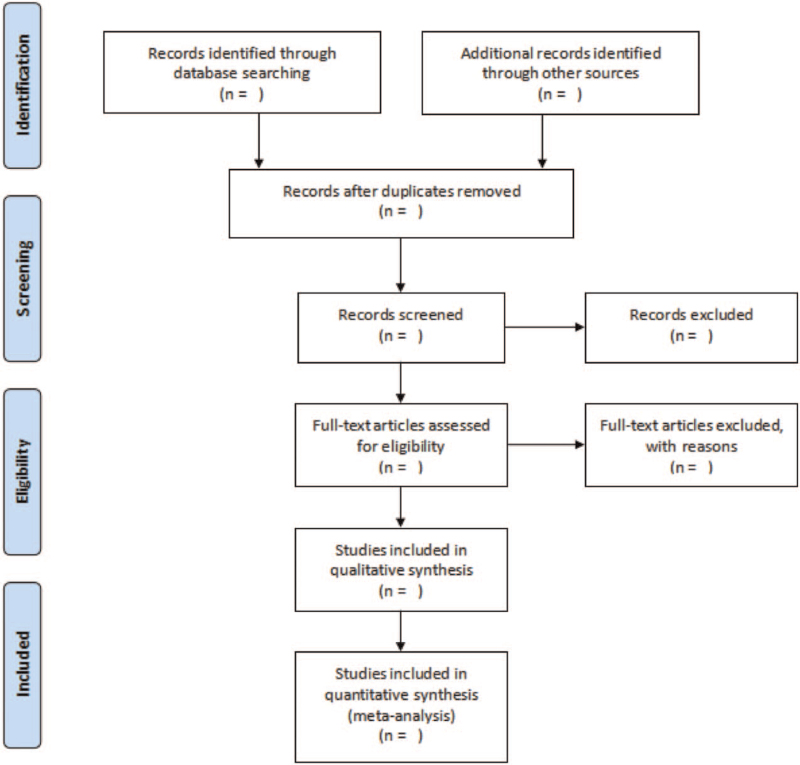
PRISMA flow diagram of the study selection process.

Two authors will independently select the trials according to the inclusion criteria, and import into Endnote X9. Then remove duplicated or ineligible studies. Screen the titles, abstracts, and full texts of all literature to identify eligible studies. All essential data will be extracted using previously created data collection sheet by 2 independent authors. Discrepancies in data collection between 2 authors will be settled down through discussion with the help of another author. The following data will be extracted from each included research: the first author's surname, publication year, language of publication, study design, sample size, number of lesions, source of the subjects, instrument, “criterion standard,” and diagnostic accuracy. The true positives, true negatives, false positives, and false negatives in the 4-fold (2 × 2) tables were also collected. Methodological quality was independently assessed by 2 researchers based on the quality assessment of studies of diagnostic accuracy studies (QUADAS) tool. The QUADAS criteria included 14 assessment items. Each of these items was scored as “yes” (2), “no” (0), or “unclear” (1). The QUADAS score ranged from 0 to 28, and a score ≥22 indicated good quality. Any disagreements between 2 investigators will be solved through discussion or consultation by a 3rd investigator.^[8]^ The specific process is illustrated in Figure 1.

#### 2.3.3 Assessment of risk of bias

Two researchers will independently evaluate the risk and bias using the Cochrane collaboration's tool. These items included in this toll will be evaluated: random sequence generation, allocation concealment, the blinding method for patients, researchers and outcomes assessors, incomplete outcome data, and selective reports. The bias risk for every item will be classed as “low risk of bias,” “high risk of bias,” “unclear risk of bias.”^[9]^

#### 2.3.4 Assessment of heterogeneity

The research will be performed by Review Manager Version 5.3 software. Heterogeneity will be evaluated by chi-squared test. If *I*^2^ value is <50%, indicating significant heterogeneity statistical results, we will use random effects model. If not, the fixed effects model, standardized mean difference, and corresponding 95% CIs will be applied for further data.

#### 2.3.5 Subgroup analysis

When there is disagreement in the results, a subgroup analysis needs to be carried out for different reasons. Heterogeneity is mainly manifested in many aspects such as race, sex, age, drug formulations, different forms of intervention, treatment time, and drug dosages.

#### 2.3.6 Data synthesis

The STATA version 14. 0 (Stata Corp, College Station, TX) and Meta-Disc version 1.4 (Universidad Complutense, Madrid, Spain) soft wares were used for meta-analysis. We calculated the pooled summary statistics for sensitivity, specificity, positive and negative likelihood ratio, and diagnostic odds ratio with their 95% confidence intervals. The summary receiver-operating characteristic curve and corresponding area under the curve were obtained. The threshold effect was assessed using Spearman correlation coefficients. The Cochrans Q-statistic and *I* test were used to evaluate potential heterogeneity between studies. If significant heterogeneity was detected (Q test *P* < .05 or *I* test >50%), a random-effects model or fixed-effects model was used. We also performed subgroup and meta-regression analyses to investigate potential sources of heterogeneity. To evaluate the influence of single studies on the overall estimate, a sensitivity analysis was performed. We conducted Beggs funnel plots and Eggers linear regression tests to investigate publication bias.

#### 2.3.7 Sensitivity analysis

When there are sufficient studies, we will carry out sensitivity analysis to test the robustness of studies according to the quality of method, the sample size, and the selection of missing data. And the fluctuation of results will be observed.

### 2.4 Ethics and dissemination

We will not obtain ethic documents because this study will be conducted basedon the data of published literature. We expect to publish this study in a peer-reviewed journal.

## 3 Discussions

The pathogenesis of CVA is similar to that of typical asthma. It is caused by chronic airway inflammation, airway hyperresponsiveness, allergen sensitization, and airway remodeling caused by immune, genetic, and environmental factors.^[10]^ Close researches over the years have found that cysteinyl leukotrienes, as important inflammatory mediators in the body, play an important role in the occurrence and development of CVA.^[11]^ CVA is not yet able to completely cure Healing can only be controlled by medication and physical therapy. The CVA treatment method widely recommended by international guidelines is inhaled low-dose glucocorticoids combined bronchodilators,^[12]^ but glucocorticoids cannot inhibit the synthesis and release of cysteinyl leukotrienes. At the same time, high-dose hormone inhalation can be absorbed from the lungs, which directly leads to systemic adverse reactions.^[13]^ to achieve the goal of clinical treatment, in some medical institutions, the treatment method of integrated traditional Chinese and western medicine is often used, and the treatment method of acupuncture combined montelukast sodium is more common. Montelukast sodium, as a selective CysLT1Rs antagonist, is recommended for combined application in patients with unsatisfactory control of simple inhaled glucocorticoid therapy. Majak et al^[14]^ researched that the leukotriene effect of CysLT1Rs antagonists can play a pharmacological effect on the inflammation caused by CysLT1 s by competing with receptors in the body.^[15]^ At present, the commonly used clinical preparations are montelukast tablets: children older than 15 years old, 10 mg/time/day, children 6 to 14 years’ old, 5 mg/time/day, children 2 to 5 years’ old, 4 mg/time/day, The drug has fewer clinical side effects and is convenient to take. Acupuncture therapy has a long history.^[16]^ It treats diseases by regulating the internal organs and meridians of the human body. It has the characteristics of simple operation, small trauma, and long-lasting effect. It is a common method for the treatment of cough variant asthma in children. Of children often have quick results. Previous studies have reported that AM can benefit for patients with CVA. However, there is no systematic review to explore this issue. Thus, this study is the first one to investigate the effectiveness and safety of AM for the treatment of patient with CVA systematically.^[17]^ The results of this study will provide helpful evidence for both clinical practice and future studies.

## Author contributions

Ye Zhang and Le Liu made similar contributions to literature retrieval and research, and wrote the first draft of the agreement.Hongshi Zhang developed a search strategy. Ye Zhang will conduct literature search and sorting. Xiaochun Feng, Ye Zhang and Xiuling Zhou will assess the risk of bias in the literature. Data analysis and article writing will be completed by Le Liu and Ye Zhang. The corresponding author Xiuling Zhou is responsible for supervising all aspects of the review and controlling the quality of the research. All authors agreed to the publish the plan.

**Conceptualization:** Xiuling Zhou, Xiaochun Feng.

**Data curation:** Ye Zhang.

**Funding acquisition:** Xiuling Zhou.

**Investigation:** Ye Zhang, Le Liu.

**Project administration:** Xiuling Zhou.

**Resources:** Hongshi Zhang.

**Supervision:** Xiuling Zhou, Hongshi Zhang.

**Validation:** Ye Zhang.

**Writing – original draft:** Ye Zhang.

**Writing – review & editing:** Ye Zhang.
